# Aging and sleep deprivation induce the unfolded protein response in the pancreas: implications for metabolism

**DOI:** 10.1111/acel.12158

**Published:** 2013-11-12

**Authors:** Nirinjini Naidoo, James G Davis, Jingxu Zhu, Maya Yabumoto, Kristan Singletary, Marishka Brown, Raymond Galante, Beamon Agarwal, Joseph A Baur

**Affiliations:** 1 Division of Sleep Medicine, Perelman School of Medicine, University of PennsylvaniaPhiladelphia, PA, USA; 2 Center for Sleep and Circadian Neurobiology, Perelman School of Medicine, University of PennsylvaniaPhiladelphia, PA, USA; 3 Institute for Diabetes, Obesity, and Metabolism, Perelman School of Medicine, University of PennsylvaniaPhiladelphia, PA, USA; 4 Department of Physiology, Perelman School of Medicine, University of PennsylvaniaPhiladelphia, PA, USA; 5 Advanced Centre for Treatment, Research and Education in Cancer (ACTREC), Tata Memorial HospitalKharghar, Navi Mumbai, India

**Keywords:** aging, behavior, glucose tolerance, mouse, sleep deprivation

## Abstract

Sleep disruption has detrimental effects on glucose metabolism through pathways that remain poorly defined. Although numerous studies have examined the consequences of sleep deprivation (SD) in the brain, few have directly tested its effects on peripheral organs. We examined several tissues in mice for induction of the unfolded protein response (UPR) following acute SD. In young animals, we found a robust induction of BiP in the pancreas, indicating an active UPR. At baseline, pancreata from aged animals exhibited a marked increase in a pro-apoptotic transcription factor, CHOP, that was amplified by SD, whereas BiP induction was not observed, suggesting a maladaptive response to cellular stress with age. Acute SD increased plasma glucose levels in both young and old animals. However, this change was not overtly related to stress in the pancreatic beta cells, as plasma insulin levels were not lower following acute SD. Accordingly, animals subjected to acute SD remained tolerant to a glucose challenge. In a chronic SD experiment, young mice were found to be sensitized to insulin and have improved glycemic control, whereas aged animals became hyperglycemic and failed to maintain appropriate plasma insulin concentrations. Our results show that both age and SD cooperate to induce the UPR in pancreatic tissue. While changes in insulin secretion are unlikely to play a major role in the acute effects of SD, CHOP induction in pancreatic tissues suggests that chronic SD may contribute to the loss or dysfunction of endocrine cells and that these effects may be exacerbated by normal aging.

## Introduction

Diabetes is a major contributor to heart disease, stroke, kidney failure, blindness, and is the seventh leading cause of death in the United States (Kochanek *et al*., 2011). Although many factors likely contribute, there is no clear consensus as to why the increase in obesity and diabetes has been so steep, or why individuals with seemingly similar risk factors go on to have dramatically different clinical outcomes. Inadequate sleep has recently been recognized as an important risk factor for insulin resistance and diabetes, and a decrease in average sleep duration in the United States has paralleled the rise in these conditions (Ayas *et al*., 2003; Gottlieb *et al*., 2005; Meisinger *et al*., 2005; Van Cauter *et al*., 2007). Large epidemiological studies also reveal an association between chronic sleep loss and obesity, which may promote insulin resistance and diabetes as secondary effects (Knutson *et al*., 2007). However, even relatively short bouts of sleep deprivation (SD) have been experimentally demonstrated to reduce glucose tolerance by as much as 40%, to suppress the acute insulin response, and to cause marked insulin resistance in peripheral tissues (Spiegel *et al*., 2005). Importantly, normal aging produces sleep disturbances, including sleep fragmentation and sleep loss in humans (Dijk *et al*., 1989, 2000; Ehlers & Kupfer, 1989; Bliwise, 1993; Prinz, 1995; Landolt *et al*., 1996) and rodents (Colas *et al*., 2005; Naidoo *et al*., 2008; Hasan *et al*., 2010). This is particularly pertinent as the aged are also at greater risk for impaired glucose tolerance and diabetes (Iozzo *et al*., 1999; Gunasekaran & Gannon, 2011; Gong & Muzumdar, 2012).

The mechanism(s) accounting for the metabolic effects of sleep deprivation are not known. One potential clue is that acute sleep deprivation induces the unfolded protein response (UPR) in the brain (Naidoo *et al*., 2005, 2008, 2011). The UPR is a coordinated adaptive response to limit the accumulation of unfolded proteins in the endoplasmic reticulum (ER) and can be induced by perturbations in calcium homeostasis, glucose/energy deprivation, redox changes, mutations that impair protein folding, or excessive secretory protein synthesis (Kaufman *et al*., 2002). Acting through three transducers – PERK, IRE1, and ATF6 – the UPR serves to reduce the number of new proteins translocated into the ER lumen, to increase retrotranslocation and degradation of ER-localized misfolded proteins, and to bolster the protein-folding capacity and secretion potential of the ER. The latter is accomplished by increasing the expression of key chaperones such as binding immunoglobulin protein (BiP) through transcriptional activity of ATF6 and another transcription factor, XBP1. The UPR further attenuates global protein translation via PERK-dependent phosphorylation of the initiation factor eIF2α. These measures work to limit protein load and alleviate ER stress. Failure to relieve ER stress leads to a maladaptive response characterized by the activation of cell death pathways, including production of the pro-apoptotic transcription factor C/EBP homologous protein (CHOP), and protein aggregation (Szegezdi *et al*., 2003, 2006).

To date, the role of the UPR in sleep deprivation has been studied only in the context of neuronal populations. However, impaired glycemic control during obesity is associated with chronic ER stress and induction of the UPR in adipose, liver, and β-cells (Hotamisligil, 2005; Ozcan *et al*., 2006). It is not known whether SD affects ER stress or the UPR in these organs. Intriguingly, BiP transcription appears to be upregulated in liver with SD (Maret *et al*., 2007), indicating that induction of the UPR may occur outside of the brain. Induction of ER stress in pancreatic β-cells can impact insulin synthesis and secretion and might therefore be expected to lead to a decrease in circulating insulin levels, with a consequent rise in blood glucose (Cnop *et al*., 2012). Excessive and/or prolonged ER stress leads to a maladaptive response and apoptosis through the activation of caspases and CHOP (Szegezdi *et al*., 2003). Insulin resistance and β-cell failure have been associated with ER stress-mediated CHOP induction in animal models (Scheuner *et al*., 2005) and humans (Laybutt *et al*., 2007; Huang *et al*., 2011). Importantly, it has been directly demonstrated that promoting the adaptive UPR (BiP) or inhibiting the maladaptive phase (CHOP) can each lead to dramatic improvements in cell survival and function (Hotamisligil, 2006, 2010). Additionally, CHOP deletion reduces oxidative stress, improves β-cell function, and promotes cell survival in multiple mouse models of diabetes (Oyadomari *et al*., 2002; Song *et al*., 2008). Several groups, including Harding *et al*. (2001), Feng *et al*. (2009), and Allagnat *et al*. (2010), have established that induction of ER stress and the UPR is sufficient to compromise insulin secretion from β-cells in a cell autonomous manner. Therefore, ER stress in the pancreas could conceivably play a major role in the effects of SD on metabolism.

In the brain, we have shown that that there is a basal level of ER stress in aged animals. Moreover, induction of the adaptive arm of the UPR by acute stresses is impaired and pro-apoptotic CHOP signaling is increased, suggesting that the UPR has already moved into the chronic/maladaptive phase (Naidoo *et al*., 2005, 2008, 2011). Consistent with this, we have shown a decline in BiP, associated with ER dyshomeostasis, during aging in the brain (Naidoo *et al*., 2008). These observations suggest that in old or obese animals, further induction of ER stress and the UPR by sleep deprivation might be much more detrimental, as cells are already primed for a maladaptive response. In this study, we examined the effect of acute sleep deprivation on glucose homeostasis and on the induction of UPR markers in peripheral tissues of young and aged mice. While most organs showed no induction of the UPR following SD, responses to both SD and aging were evident in the pancreas. Thus, we hypothesized that aged animals, which display molecular markers of ER stress under basal conditions, might be susceptible to further stress resulting from SD. In fact, we observed that young animals were able to metabolically respond to SD very well, while aged control animals exhibited a loss of glycemic control. These observations have important implications, as many sleep studies in humans are performed on young individuals who may be more tolerant of the stress than older individuals.

## Results

### Sleep deprivation induces UPR in peripheral tissues

**Figure 1:**
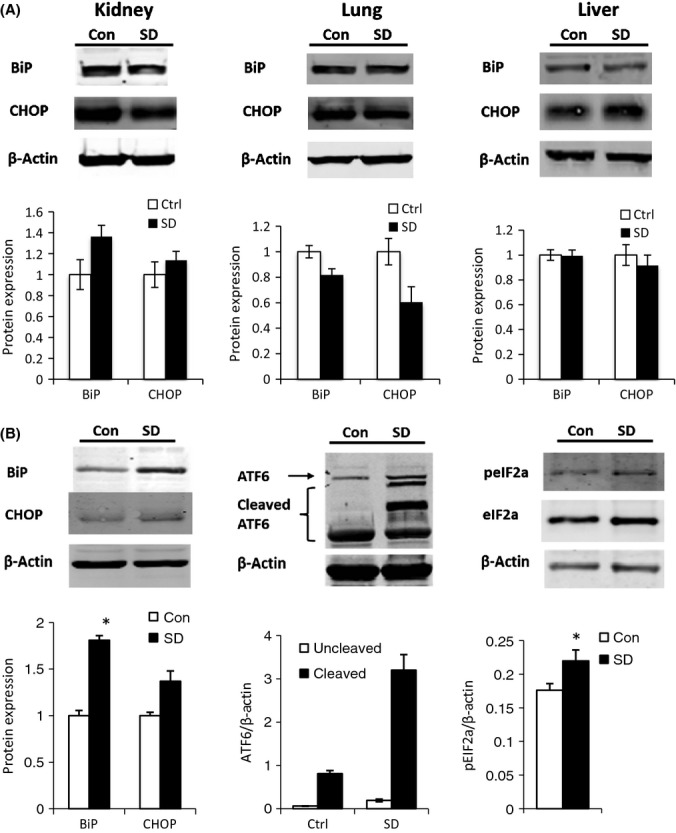
SD induces UPR outside of the CNS. Acute sleep deprivation induces the UPR outside of the CNS. Peripheral tissue from sleep-deprived (*n* = 6) and undisturbed (*n* = 6) mice were surveyed for markers of the adaptive (BiP) and apoptotic arms (CHOP) of the UPR. (A) Representative Westerns from kidney, lung, and liver tissue show BiP and CHOP expression in the respective tissues. Histograms show BiP and CHOP protein quantification after normalization to β-actin in each tissue; average expression ± SEM shown. There is no significant increase in BiP or CHOP protein in these tissues with sleep deprivation. (B) Sleep deprivation induces the UPR in pancreas. Representative Westerns of BiP, CHOP, cleaved ATF6, and P-eIF2α in pancreatic lysates of young sleep-deprived (SD) mice for 6 h and in undisturbed (CON) mice *n* = 8/group). Graphs below Westerns illustrate the quantification of each of the UPR markers; average ± SEM shown. BiP expression is significantly increased (*P = 0.001) with sleep deprivation. ATF6 cleavage is increased and phosphorylation of eIF2α is also significantly increased (*n* = 8; *P = 0.04) with SD.

Sleep deprivation leads to endoplasmic reticulum stress in the brain, resulting in the induction of the unfolded protein response. We surveyed additional tissues from sleep-deprived animals for the induction of BiP and CHOP, key components of the unfolded protein response (Hetz, 2012). Peripheral tissues (liver, lung, kidney, and pancreas) from sleep-deprived (*n* = 6) and undisturbed (*n* = 6) mice were surveyed for markers of the adaptive (BiP) and apoptotic arms (CHOP) of the UPR. No induction of these proteins was noted in liver, lung, or kidney from SD mice (Fig. 1A); however, BiP expression was significantly increased (*P = 0.001) with sleep deprivation in the pancreas (Fig. 1B) in each of three independent experiments. Induction of the UPR was confirmed using additional markers, including cleavage of ATF6 and phosphorylation of eukaryotic initiation factor 2α (eIF2α), both of which were significantly increased with SD (Fig. 1B). Expression of CHOP also trended higher, but exhibited a high degree of variation between individuals.

### Age and SD induce UPR in pancreas tissue

**Figure 2:**
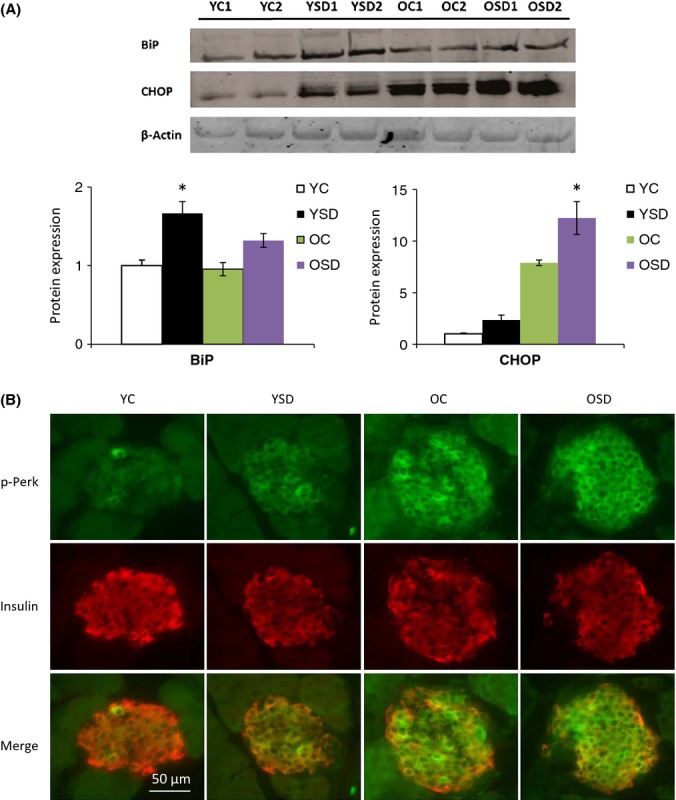
Pancreatic UPR becomes maladaptive in older animals. (A) CHOP expression is increased when aged mice are challenged with sleep deprivation. Young (3 months) and aged (24 months) mice were subjected to 6 h of SD. Western blots of pancreatic tissue indicate that there is a significant increase in BiP in young (*P = 0.001) and not in aged pancreas with SD and significantly more CHOP expressed in the aged mice at baseline that is further increased with SD (*P < 0.01). Gel shows BiP and CHOP protein in young control (YC) and SD (YSD) and in old control (OC) and old SD (OSD) probed on the same gel. Bar graph shows BiP and CHOP expression normalized to β-actin (average ± SEM) in young and aged mice from four gels (*n* = 8). (B) There is increased ER stress in the pancreas of aged mice. Phosphorylation of PERK (P-PERK), indicative of ER stress, and activation of the unfolded protein response are evident in β-cells. Representative images of P-PERK (green) in B cells (red) immunostained for insulin in young undisturbed (YC), young SD (YSD), old undisturbed (OC), and old sleep-deprived (OSD) mice.

The induction of the UPR in response to acute SD in the brain exhibits age-related defects (Naidoo *et al*., 2008). To test whether this is true in peripheral tissues, we subjected aged (22–27 month-old) mice to the same SD regimen and measured pancreatic BiP and CHOP expression. Western blots of pancreatic tissue indicate that there is a significant increase in BiP in young (*P = 0.001) and not in aged pancreas with SD (Fig. 2A). At the same time, we observed significantly more CHOP expressed in the aged mice at baseline, which is further increased by SD (*P < 0.01).

The bulk of the pancreas is composed of exocrine tissue. The endocrine cells located within the islets of Langerhans, and in particular, the insulin-secreting β-cells, play a major role in determining whole-body glucose homeostasis. To directly test whether β-cells exhibit UPR induction, we stained sectioned pancreata with an antibody to phosphorylated PERK (protein kinase RNA-like endoplasmic reticulum kinase). PERK is a serine threonine transmembrane kinase that is responsible for repressing protein synthesis. PERK is activated via autophosphorylation upon dissociation from BiP, which occurs when BiP is recruited to chaperone-misfolded proteins. Activated PERK phosphorylates eIF2α leading to a stalled initiation complex, inhibition of protein translation, and hence, reduced protein load and ER stress (Kaufman *et al*., 2002). Phosphorylated PERK was detected in β-cells from each of the treatment groups and was markedly higher in aged animals (Fig. 2B). These findings indicate that endocrine cells are among the cell types within the pancreas that exhibit ER stress and induction of the UPR.

### SD affects glucose homeostasis

**Figure 3:**
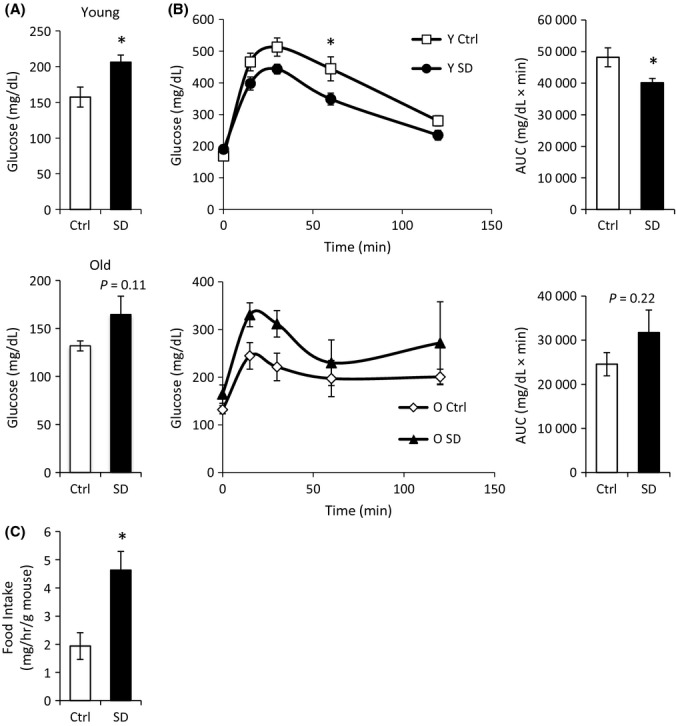
Acute sleep deprivation increases glucose and impairs glucose tolerance in older animals. (A) Mice subjected to 6 h of sleep deprivation had higher blood glucose levels [*n* = 6 Ctrl, 6 SD (young), 5 Ctrl, 4 SD (old)]. (B) In young mice (Y, top, *n* = 6 Ctrl, 6 SD), 6 h of prior sleep deprivation moderately improved glucose clearance in a glucose tolerance test (2 g kg^−1^ i.p.), whereas older mice subjected to sleep deprivation (O, bottom, *n* = 5 Ctrl, 4 SD) exhibited a clear trend toward an impairment in glucose tolerance. AUC – area under the glucose curve. (C) Sleep deprivation increases food intake (*n* = 8/group). All error bars show SEM, *P < 0.05.

Next, we asked whether acute SD was sufficient to alter glucose homeostasis. Blood glucose levels were significantly higher in animals subjected to SD as compared to controls (Fig. 3A). However, glucose tolerance was improved in young animals following SD (Fig. 3B). This result contrasts with our findings in older animals, where glucose tolerance tended to be worse following SD. Because SD affords a greater opportunity for the mice to eat, we wondered whether food intake might be greater in the SD group and whether this might be influencing our results. Indeed, food intake over the 6-h period of SD was more than double in the SD group compared with controls that were allowed to sleep *ad libitum* (Fig. 3C).

### Acute SD increases glucose, but does not affect glucose tolerance in the absence of food

**Figure 4:**
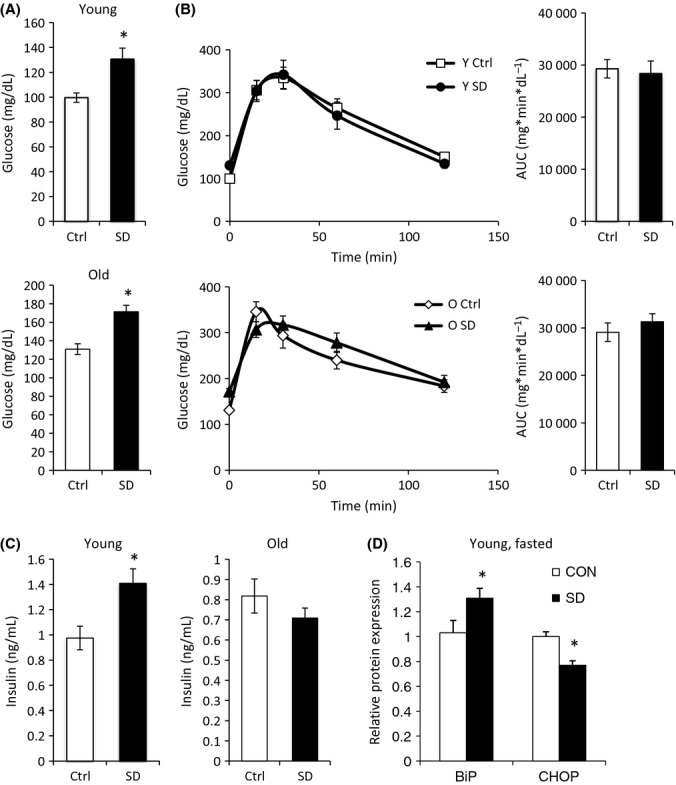
Changes in blood glucose, but not glucose tolerance, following sleep deprivation are independent of food intake. (A) Young or old mice subjected to 5 h of sleep deprivation in the absence of food had higher blood glucose. (B) Glucose tolerance of young mice (Y, top, *n* = 8 Ctrl, 8 SD) or old (O, bottom, *n* = 15 Ctrl, 17 SD) mice is not significantly affected by sleep deprivation in the absence of food. A total of 2 g kg^−1^ glucose was injected after 5 h of sleep deprivation. Sixty minutes corresponds to the end of sleep deprivation. AUC – area under the glucose curve. (C) Insulin levels are higher in young animals and unchanged in old animals subjected to acute sleep deprivation. (D) The effect of sleep deprivation on BiP in the pancreas is independent of food, but the CHOP response is reversed in young fasting animals. CHOP and β-actin in sleep-deprived and undisturbed mice pancreatic lysates from Western blots. Graph illustrates the decrease in CHOP expression in pancreas following sleep deprivation (*n* = 8). All error bars show SEM, *P < 0.05.

To determine the influence of SD in the absence of confounding differences in food intake, we repeated the SD experiments in young and aged mice that were fasted throughout the experiment. Interestingly, the increased blood glucose levels following SD were preserved (Fig. 4A). However, the improvement in glucose tolerance in the young mice and worsening of glucose tolerance in aged mice were prevented, suggesting that they were due to the confounding influence of food intake (Fig. 4B). Notably, glucose tolerance was impaired in one cohort of aged animals, but the effect disappeared when data from several experiments were combined. This may reflect heterogeneity among aged individuals in the response to acute sleep loss. One potential mechanism to explain the higher glucose in animals subjected to SD is that inhibition of protein translation due to ER stress and PERK activation compromises insulin secretion. However, we were not able to detect a significant decrease in insulin following acute SD in any experiment. In contrast, we found that on average, insulin was higher in young animals subjected to SD (Fig. 4C), although the effect was not apparent in every experiment. In the absence of food, BiP was still induced following SD (*P* = 0.03, *n* = 7), whereas CHOP induction was completely prevented; instead, CHOP expression was significantly lower following SD (*P* < 0.01; Fig. 4D).

### Corticosterone, glucose, and insulin levels

**Figure 5:**
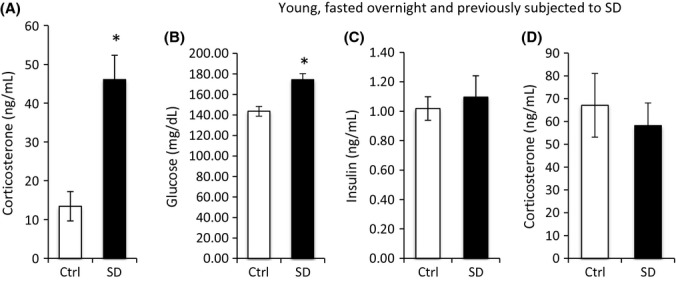
Corticosterone is increased by acute sleep deprivation. (A) Young mice subjected to 6 h of sleep deprivation in the presence of food had increased corticosterone levels as compared to controls measured at the same time point (1 PM; *n* = 9/group). (B–D) Mice that were previously subjected to sleep deprivation were allowed to recover for 1 week and then fasted overnight and subjected to sleep deprivation in the absence of food. Blood glucose was higher (B) in the absence of changes in insulin (C) or corticosterone (D) (*n* = 5/group). All error bars show SEM, *P < 0.05.

We measured circulating corticosterone levels to determine whether a hormonal stress response may be contributing to SD-induced insulin resistance. In a large independent cohort of young animals subjected to SD in the presence of food, corticosterone levels measured immediately following the treatment period were robustly increased above the values obtained for control animals (Fig. 5A). Similar results were obtained from older, fasted animals (Ctrl – 40 ± 10, SD – 107 ± 14 ng mL^−1^, *P* < 0.003). These results suggest that stress hormones such as corticosterone might play a role in the response to acute SD. However, the corticosterone concentrations observed in SD animals were within the stated ‘normal’ range of the assay (47–159 ng mL^−1^, Corticosterone Mouse/Rat ELISA, Alpco Diagnostics, Salem, NH, USA) and below those typically induced by deliberate stress [e.g., 450–500 ng mL^−1^ reported by Flint and Tinkle for a 2 h restraint stress (Flint & Tinkle, 2001)], raising the question of whether they would be sufficient to produce major changes in glucose homeostasis. In support of the idea that glucose changes are at least partially independent of corticosterone, mice that were subjected to SD, allowed to recover for 1 week, and then subjected to SD again after an overnight fast displayed increased glucose with no changes in corticosterone or insulin (Fig. 5B–D). However, we cannot rule out the possibility that the corticosterone levels in our studies may have spiked earlier during SD and then stabilized by the 6-h time point, when the measurements were made.

### Chronic SD has age-dependent effects on glucose metabolism

**Figure 6:**
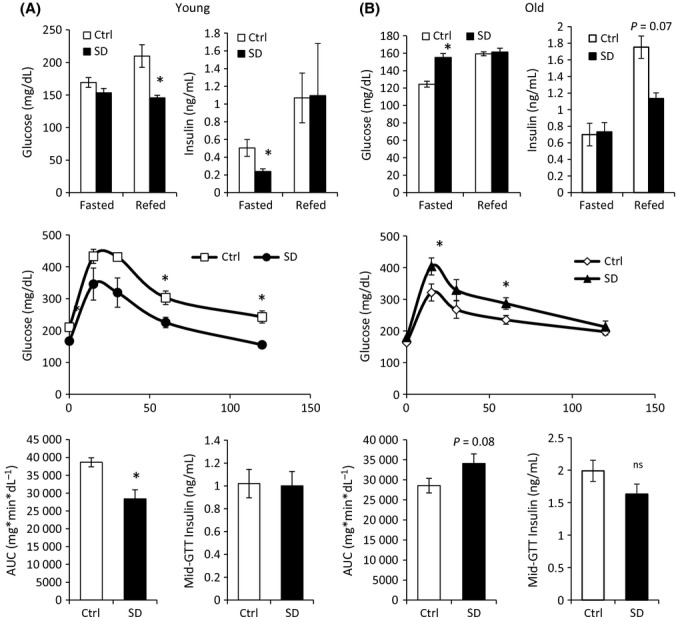
Chronic sleep deprivation has age-dependent effect on glucose metabolism. (A) Young (3 months) mice subjected to sleep deprivation 20 h per day for 8 days had lower plasma glucose levels following refeeding and during a glucose tolerance test. Insulin levels were low or normal under all conditions studied. Food was removed 4 h prior to the start of the GTT (*n* = 4 Ctrl, 4 SD for GTT, 7 Ctrl, 8 SD for all other measures). (B) Aged (22–24 months) mice subjected to sleep deprivation 20 h per day for 8 days had higher plasma glucose levels when fasting and during a glucose tolerance test. Despite increased glucose, insulin levels trended lower after refeeding and during the GTT (*n* = 8 Ctrl, 7 SD). All error bars show SEM, *P < 0.05.

Because our results suggested that the pancreas was able to function normally in the setting of acute SD, we next examined the effects of chronic (8 day) sleep restriction, which may be more relevant to human health. Mice were placed in automated sleep deprivation chambers (Pinnacle Technologies) to restrict sleep for 20 h of each day (Hines *et al*., 2013). Interestingly, this model revealed a sharp contrast between the responses of young and aged mice. In young animals, glucose levels were consistently lower following SD (Fig. 6A). Insulin levels were also reduced under fasting conditions, suggesting improved insulin sensitivity, but were readily induced by refeeding or glucose challenge. Total area under the curve for the glucose tolerance test was significantly lower in young mice subjected to chronic SD. On the other hand, aged mice subjected to chronic SD had higher fasting glucose levels and tended to have lower insulin following refeeding or glucose challenge, despite significant increases in blood glucose (Fig. 6B). In contrast to our findings in young animals, these results provide evidence that chronic SD in aged animals decreases peripheral insulin sensitivity and leads to inappropriately low insulin secretion, both of which may contribute to the degradation of glycemic control.

## Discussion

Overall, our results show that both SD and aging place stress on the pancreas and support the notion that in a chronic setting, the combination may lead to defects in insulin secretion that contribute to the diabetogenic effects of sleep loss. These findings highlight the importance of considering age as a variable in studies of metabolism and provide further evidence that acute and chronic sleep loss provoke distinct responses. Determining whether ER stress and the UPR are causal in, or simply correlated with, the effects of SD on insulin secretion will be an important next step.

We have previously shown that sleep deprivation leads to ER stress in the brain, resulting in the induction of the unfolded protein response. Given the emerging association between sleep disruption and diabetes, and the potential roles for ER stress in contributing to diabetic phenotypes, we surveyed the liver and pancreatic tissues from sleep-deprived animals for the induction of BiP and CHOP, key components of the unfolded protein response (Hetz, 2012). We found that SD increased expression of BiP, phosphorylation of eIF2α, and cleavage of ATF6 in the pancreata of young mice, indicating upregulation of the adaptive arm of the UPR. However, the acute induction of BiP was lost in aged animals, while CHOP was markedly increased in the pancreata of aged mice under both undisturbed and sleep-deprived conditions. This is consistent with our earlier studies indicating that there is increased/basal ER stress in the brain of aged mice (Naidoo *et al*., 2008, 2011). Although the bulk of the pancreas is exocrine tissue, phospho-PERK immunofluorescence experiments confirmed the existence of ER stress in insulin-secreting β-cells, suggesting that SD might have consequences for endocrine function. Overall, these results are consistent with a maladaptive response to ongoing ER stress in pancreata from aged animals that might be exacerbated by SD.

To test the hypothesis that ER stress induced by SD would impair endocrine function, we next explored the effects of acute SD on glucose homeostasis in mice. While our standard SD protocol modestly raised initial glucose levels in both young and old mice, it had divergent effects on their performances in a glucose tolerance test; young mice were more effective at clearing glucose following SD, while glucose values in old mice tended to remain higher. The observation that mice subjected to SD eat significantly more than controls during the intervention (Fig. 3C) provides a potential explanation for both the increased glucose and the improved glucose tolerance in young SD mice. Sequential glucose loads result in improved glucose tolerance (the Staub–Traugott effect) due to the factors that include potentiation of insulin secretion and suppression of hepatic glucose output (Bonuccelli *et al*., 2009). Therefore, increased food consumption in the SD mice might have influenced our observations and might account for a subsequent improvement in glucose tolerance. While this highlights one potential benefit of an acute increase in food consumption, our observations also suggest the potential for sustained overconsumption of food in the setting of chronic SD, which could contribute to weight gain and the development of diabetes.

When young and old mice were subjected to SD in the absence of food, the increase in initial glucose levels persisted, whereas the improvement in glucose tolerance in younger animals was eliminated, consistent with it being attributable to the Staub–Traugott effect. Contrary to our hypothesis that changes in glucose homeostasis would be driven by endocrine dysfunction, the increases in glucose following SD were not associated with any decrease in circulating insulin levels. In fact, insulin was significantly increased by SD in younger animals in some of our experiments, possibly secondary to the change in glucose. This suggests that glucose is higher during acute SD primarily due to insulin resistance of unknown etiology, rather than to a defect in insulin secretion related to pancreatic ER stress. An important limitation of this study was that only wild-type animals were employed. In the future, it will be very interesting to use genetic models and/or chemical chaperones to directly test the role of the pancreatic UPR in whole-body glucose phenotypes.

Interestingly, removing food during SD uncoupled its effects on BiP and CHOP. BiP induction was preserved in the absence of food, whereas CHOP expression was significantly decreased following SD. This suggests that either BiP and CHOP are induced by discrete stimuli during SD or CHOP induction has a higher threshold in terms of the intensity or duration of the stimulus. One possible interpretation of these findings is that BiP induction reflects a protective response that could increase protein-folding capacity, whereas CHOP induction is a direct reflection of ER stress induced by factors related to food intake.

We next considered whether a hormonal stress response might be contributing to SD-induced insulin resistance. Notably, studies in adrenalectomized mice indicate that the UPR is turned on by SD in the brain independently from corticosterone (Mongrain *et al*., 2010); however, whether this is also true in the pancreas or whether glucocorticoids have a major role in glucose homeostasis during SD are not known. Corticosterone levels were robustly increased above the values obtained for control animals (Fig. 5A), yet remained below those typically induced by deliberate stress (Flint & Tinkle, 2001), making it unclear whether they would be sufficient to cause detectable changes in glucose homeostasis. Moreover, when mice that had previously experienced SD were fasted overnight to normalize hormone levels and then subjected to SD in the absence of food, we detected a clear increase in blood glucose with no change in plasma insulin or corticosterone levels, indicating that neither of these can fully account for the effects of acute SD on glucose homeostasis. These observations are consistent with reports that sleep deprivation decreases insulin sensitivity without changes in cortisol levels in healthy humans (Gonzalez-Ortiz *et al*., 2000; van Leeuwen *et al*., 2009). We note, however, that even small changes in corticosterone levels may contribute to the long-term effects of sleep loss, and we cannot rule out a rapid spike in corticosterone levels earlier in the acute sleep deprivation protocol as a contributing factor in glucose dyshomeostasis.

To test whether a more chronic model of SD would lead to impaired β-cell function, we subjected young and old mice to 8 days of SD for 20 h per day. In this setting, the responses of young and old mice were clearly divergent, with young animals showing improved glucose homeostasis, while aged animals became hyperglycemic and displayed inappropriately low levels of plasma insulin. Dissecting out these mechanisms may have important implications for humans who suffer from inadequate sleep, many of whom are older and may not be accurately modeled based on studies conducted in younger individuals. A comparable study in humans (1 week with sleep restricted to 5 h per night) revealed impaired glycemic control and insulin resistance (Buxton *et al*., 2010), suggesting that the older mice might better reflect the effects of SD in humans. While plasma insulin levels were normal in the human subjects, the authors note that there was no compensatory increase in insulin secretion despite insulin resistance, which might be an early indication of β-cell dysfunction. In support of the idea that impaired insulin secretion is a contributing factor to the loss of glycemic control during chronic SD, Barf *et al*. (2010) reported that insulin levels decline precipitously after 8 days of SD in rats, whereas acute treatment has no effect. Taken together, these studies support the idea that SD has pronounced effects on insulin sensitivity, but in a chronic setting, may also lead to impaired insulin secretion, both of which could be contributing factors in the onset of diabetes and metabolic syndrome in humans.

A critical goal for future work will be to determine whether ER stress and the UPR underlie the effects of chronic SD on plasma insulin levels. One potential mechanism that could account for such an effect was recently described by Fonseca *et al*. (2012), who showed that ER stress prevents Wolfram syndrome 1 (WFS1) from properly localizing to the plasma membrane to facilitate insulin secretion following glucose stimulation in β-cells. Intriguingly, inactivation of the XBP1-dependent arm of the UPR leads to impaired processing and secretion of insulin, which may result from compensatory upregulation of IRE1α, another key component of the UPR that targets the mRNAs of proinsulin-processing enzymes for degradation (Lee *et al*., 2011). Although the effects of ER stress on insulin secretion can be modeled in cultured cells (Laybutt *et al*., 2007; Pirot *et al*., 2007; Feng *et al*., 2009; Ota & Wang, 2012), there is no obvious way to mimic SD in this model, meaning that elucidating the mechanism for SD effects will require studies in intact animals. *In vivo*, interpretation of experiments involving ER stress can be quite challenging to interpret. For instance, dosing animals 4-phenyl butyrate (PBA) to protect against ER stress has a strong glucose-lowering effect even under basal conditions (Ozcan *et al*., 2006) Overexpression of BiP in β-cells has been shown to protect against apoptosis and diabetes progression in db/db mice (Laybutt *et al*., 2007), and it would be interesting to examine the effects of chronic SD in this model. Moreover, Song *et al*. (2008) have reported that deleting CHOP from β-cells is sufficient to promote cell survival and prevent diabetes during metabolic stress. Given that β-cell dysfunction is generally considered the event that tips the balance from insulin resistance to overt diabetes in humans, these observations may be directly related to the association between sleep disruption and diabetes in epidemiological studies. Our findings suggest that age and SD cooperate to induce chronic ER stress that could lead to the death or dysfunction of β-cells, and ultimately exacerbate metabolic dysfunction by compromising insulin secretion.

## Experimental procedures

### Animals

Studies were performed on young 10-week and aged 22- to 27-month-old C57BL/6 male mice maintained on a 12:12 light–dark cycle (lights on at 7 AM). Mice were obtained from Jackson Labs with the exception of the aged animals, which were obtained from the National Institute on Aging Aged Rodent Colony. Animals were kept in individual cages in a sound-attenuated room with ambient temperature 23.5 ± 1.0 °C, humidity 40% ± 5%, and light intensity of 50–60 lux measured at the level of the mice with water and food available *ad libitum*. Mice were allowed to acclimate to their new environment for at least 2 weeks before starting any experiments. Animal handling and experimental procedures followed the National Institutes of Health Guide for the Care and Use of Laboratory Animals and were approved by the Animal Care and Use Committee of the University of Pennsylvania.

### Sleep deprivation

Sleep deprivation was initiated at lights on (7.00 AM), and deprivation for 6 h was performed through gentle handling as previously described (Cirelli & Tononi, 1998; Naidoo *et al*., 2005). All the animals in the study were acclimated for at least 5 days prior to studies and were adapted to handling procedures prior to the experiment. Briefly, sleep deprivation included directly observing the animal’s motor activity and gently stroking the fur with an artist’s brush when no activity was observed.

### Chronic sleep deprivation

Young and aged mice were subjected to chronic sleep deprivation for 20 h per day from 7 PM to 3 PM for 7 days using the Automated Sleep Deprivation System for Mice (Pinnacle technologies; Hines *et al*., 2013). All the animals in the study were acclimated to the sleep deprivation chambers for at least 5 days prior to studies.

### Food intake

To assess the effect of acute sleep deprivation on food intake, food pellets in each cage were weighed before and after a 6-h study that included both sleep-deprived and undisturbed mice.

### Tissue preparation

Fresh or frozen pancreatic tissue was sonicated on ice in a lysis buffer (20 mm Tris–HCl pH 7.5, 1 mm EGTA, 1 mm EDTA, 1% Triton X-100, 10% glycerol) in the presence of protease (1 mm PMSF, 2 μg mL^−1^ pepstatin, and 4 μg mL^−1^ aprotinin) and phosphatase inhibitors (1 mm orthovanadate). The lysate was centrifuged at 16 000 *g* for 10 min, and the supernatant was collected. Protein was determined by the Pierce micro-BCA assay.

### Western blots

Individual sleep-deprived and matching control mice pancreatic homogenates were run on SDS-PAGE gels in duplicates or triplicates. Samples (20 μg protein) representing individual mice were run on SDS-PAGE (Bio-Rad, 10% Tris–HCl) according to Laemmli (1970) and then transferred to nitrocellulose membranes (Bio-Rad). Following transfer onto nitrocellulose, blots were incubated with primary antibody (see list below for primary antibody and dilution). After incubation with secondary antibody, protein bands were detected and analyzed by enhanced chemiluminescence (Pierce Supersignal) and quantitative imaging (AlphaInnotech Fluorochem 8900). Densitometry was performed using the Alphaease FC software. Alternatively, IR-conjugated secondary antibodies were used, and protein bands were detected and quantified by infrared imaging on an Odyssey (LiCor).

**Table:** 

Antibody	Dilution	Company	Cat no.	Animal
BiP/GRP78	1:1000	Enzo Life Sciences	ADI-SPA-826	Polyclonal rabbit
CHOP/GADD153	1:500	Santa Cruz Biotech	sc-575	Polyclonal rabbit
peif2α (Ser51) (119A11)	1:1000	Cell Signaling	3597S	Monoclonal rabbit
ATF6	1:1000	Imgenex	IMG-273	Monoclonal mouse
β-actin	1:1000	Cell Signaling	3700S	Monoclonal mouse

### Immunohistochemistry

Tissues were fixed in 4% paraformaldehyde overnight at 4 °C and then transferred to 30% sucrose for cryoprotection. Cryoprotected tissues were embedded in Tissue-Tek embedding medium (OCT compound, Sakura Finetek Inc., Torrance, CA, USA) on dry ice. These tissue blocks were stored at −80 °C. Ten-micrometer cryostat sections were collected directly onto slides with a permanent positively charged surface (Superfrost/Plus Slides, Cat # 12-550-15; Fisher Scientific, Hampton, NH, USA). Slides were stored at −80 °C until immunohistochemistry staining was performed. Antigen retrieval (microwaving) was performed in Tris–EDTA buffer (10 mm Tris base, 1 mm EDTA solution, 0.05% Tween-20, pH 9.0) for 20 min at 95 °C. After blocking the sections with blocking buffer (4% normal donkey serum, 1% BSA, 0.4% Triton X-100 in 1× PBS), slides were incubated with primary antibodies against p-PERK (Thr 981; rabbit; 1:100 dilution, sc-32577; Santa Cruz Biotechnology, Santa Cruz, CA, USA) and insulin (guinea pig; 1:3000 dilution, ab7842; Abcam, Cambridge, MA, USA) overnight at 4 °C diluted in blocking buffer. The following day, slides were washed with PBS, incubated with donkey secondary antibodies (donkey anti-rabbit Alexa Fluor-488 and donkey anti-guinea pig Alexa Fluor-594 at 1:500 dilution) for 90 min at room temperature, washed six times with PBS (5 min each), and then mounted with a SlowFade Gold antifade mounting medium with DAPI (Cat No. S36938; Invitrogen, Carlsbad, CA, USA).

### Glucose, insulin, and corticosterone measurements

Blood was collected for plasma preparation in heparinized tubes. Glucose readings were taken from whole blood using a glucometer (OneTouch Ultra; LifeScan Inc., Milpitas, CA, USA). Mouse insulin was measured in plasma by ELISA using the ultrasensitive kit from ALPCO Diagnostics (Salem). Mouse corticosterone was measured in plasma using a radioimmunoassay from MP Biochemicals (ImmuChem double antibody Corticosterone ^125^I RIA kit; Orangeburg, NY, USA) (Fig. 5A) or an ELISA from ALPCO Diagnostics (Salem; older animals described in the text and Fig. 5D).

### Glucose tolerance tests

Experimental and control age- and weight-matched wild-type C57BL/6 mice, either young (3–6 months) or aged (22–27 months), were singly housed. Sleep deprivation was accomplished as described above. Animals were injected intraperitoneally with glucose at 2 g kg^−1^ bodyweight at 5 or 6 h of sleep deprivation, as indicated, and glucose values were monitored in tail blood with a glucometer (OneTouch Ultra; LifeScan Inc.). Glucose values were measured at baseline, 15, 30, 60, and 120 min after injection. Where indicated, mice were also subjected to fasting starting at 7 AM, concurrent with sleep deprivation. One mouse was removed from the old control group in Fig. 4B due to a moribund appearance and immobility on the day of the experiment.

## References

[b51] Allagnat F, Christulia F, Ortis F, Pirot P, Lortz S, Lenzen S, Eizirik DL, Cardozo AK (2010). Sustained production of spliced X-box binding protein 1 (XBP1) induces pancreatic beta cell dysfunction and apoptosis. Diabetologia.

[b1] Ayas NT, White DP, Al-Delaimy WK, Manson JE, Stampfer MJ, Speizer FE, Patel S, Hu FB (2003). A prospective study of self-reported sleep duration and incident diabetes in women. Diabetes Care.

[b2] Barf RP, Meerlo P, Scheurink AJ (2010). Chronic sleep disturbance impairs glucose homeostasis in rats. Int. J. Endocrinol.

[b3] Bliwise DL (1993). Sleep in normal aging and dementia. Sleep.

[b4] Bonuccelli S, Muscelli E, Gastaldelli A, Barsotti E, Astiarraga BD, Holst JJ, Mari A, Ferrannini E (2009). Improved tolerance to sequential glucose loading (Staub–Traugott effect): size and mechanisms. Am. J. Physiol. Endocrinol. Metab.

[b5] Buxton OM, Pavlova M, Reid EW, Wang W, Simonson DC, Adler GK (2010). Sleep restriction for 1 week reduces insulin sensitivity in healthy men. Diabetes.

[b6] Cirelli C, Tononi G (1998). Changes in anti-phosphoserine and anti-phosphothreonine antibody binding during the sleep-waking cycle and after lesions of the locus coeruleus. Sleep Res. Online.

[b7] Cnop M, Foufelle F, Velloso LA (2012). Endoplasmic reticulum stress, obesity and diabetes. Trends Mol. Med.

[b8] Colas D, Cespuglio R, Sarda N (2005). Sleep wake profile and EEG spectral power in young or old senescence accelerated mice. Neurobiol. Aging.

[b9] Dijk DJ, Beersma DG, van den Hoofdakker RH (1989). All night spectral analysis of EEG sleep in young adult and middle-aged male subjects. Neurobiol. Aging.

[b10] Dijk DJ, Duffy JF, Czeisler CA (2000). Contribution of circadian physiology and sleep homeostasis to age-related changes in human sleep. Chronobiol. Int.

[b11] Ehlers CL, Kupfer DJ (1989). Effects of age on delta and REM sleep parameters. Electroencephalogr. Clin. Neurophysiol.

[b12] Feng D, Wei J, Gupta S, McGrath BC, Cavener DR (2009). Acute ablation of PERK results in ER dysfunctions followed by reduced insulin secretion and cell proliferation. BMC Cell Biol.

[b13] Flint MS, Tinkle SS (2001). C57BL/6 mice are resistant to acute restraint modulation of cutaneous hypersensitivity. Toxicol. Sci.

[b14] Fonseca SG, Urano F, Weir GC, Gromada J, Burcin M (2012). Wolfram syndrome 1 and adenylyl cyclase 8 interact at the plasma membrane to regulate insulin production and secretion. Nat. Cell Biol.

[b15] Gong Z, Muzumdar RH (2012). Pancreatic function, type 2 diabetes, and metabolism in aging. Int. J. Endocrinol.

[b16] Gonzalez-Ortiz M, Martinez-Abundis E, Balcazar-Munoz BR, Pascoe-Gonzalez S (2000). Effect of sleep deprivation on insulin sensitivity and cortisol concentration in healthy subjects. Diabetes Nutr. Metab.

[b17] Gottlieb DJ, Punjabi NM, Newman AB, Resnick HE, Redline S, Baldwin CM, Nieto FJ (2005). Association of sleep time with diabetes mellitus and impaired glucose tolerance. Arch. Intern. Med.

[b18] Gunasekaran U, Gannon M (2011). Type 2 diabetes and the aging pancreatic beta cell. Aging (Albany NY).

[b52] Harding HP, Zeng H, Zhang Y, Jungries R, Chung P, Plesken H, Sabatini DD, Ron D (2001). Diabetes mellitus and exocrine pancreatic dysfunction in perk-/- mice reveals a role for translational control in secretory cell survival. Mol. Cell.

[b19] Hasan S, Dauvilliers Y, Mongrain V, Franken P, Tafti M (2010). Age-related changes in sleep in inbred mice are genotype dependent. Neurobiol. Aging.

[b20] Hetz C (2012). The unfolded protein response: controlling cell fate decisions under ER stress and beyond. Nat. Rev. Mol. Cell Biol.

[b21] Hines DJ, Schmitt LI, Hines RM, Moss SJ, Haydon PG (2013). Antidepressant effects of sleep deprivation require astrocyte-dependent adenosine mediated signaling. Transl. Psychiatry.

[b22] Hotamisligil GS (2005). Role of endoplasmic reticulum stress and c-Jun NH2-terminal kinase pathways in inflammation and origin of obesity and diabetes. Diabetes.

[b23] Hotamisligil GS (2006). Inflammation and metabolic disorders. Nature.

[b24] Hotamisligil GS (2010). Endoplasmic reticulum stress and the inflammatory basis of metabolic disease. Cell.

[b25] Huang W, Ramsey KM, Marcheva B, Bass J (2011). Circadian rhythms, sleep, and metabolism. J. Clin. Invest.

[b26] Iozzo P, Beck-Nielsen H, Laakso M, Smith U, Yki-Jarvinen H, Ferrannini E (1999). Independent influence of age on basal insulin secretion in nondiabetic humans. European Group for the Study of Insulin Resistance. J. Clin. Endocrinol. Metab.

[b27] Kaufman RJ, Scheuner D, Schroder M, Shen X, Lee K, Liu CY, Arnold SM (2002). The unfolded protein response in nutrient sensing and differentiation. Nat. Rev. Mol. Cell Biol.

[b28] Knutson KL, Spiegel K, Penev P, Van Cauter E (2007). The metabolic consequences of sleep deprivation. Sleep Med. Rev.

[b29] Kochanek KD, Xu J, Murphy SL, Minino AM, Kung HC (2011). Deaths: final data for 2009. Natl. Vital Stat. Rep.

[b53] Laemmli UK (1970). Cleavage of structural proteins during the assembly of the head of bacteriophage T4. Nature.

[b30] Landolt HP, Dijk DJ, Achermann P, Borbely AA (1996). Effect of age on the sleep EEG: slow-wave activity and spindle frequency activity in young and middle-aged men. Brain Res.

[b31] Laybutt DR, Preston AM, Akerfeldt MC, Kench JG, Busch AK, Biankin AV, Biden TJ (2007). Endoplasmic reticulum stress contributes to beta cell apoptosis in type 2 diabetes. Diabetologia.

[b32] Lee AH, Heidtman K, Hotamisligil GS, Glimcher LH (2011). Dual and opposing roles of the unfolded protein response regulated by IRE1alpha and XBP1 in proinsulin processing and insulin secretion. Proc. Natl Acad. Sci. USA.

[b33] van Leeuwen WM, Lehto M, Karisola P, Lindholm H, Luukkonen R, Sallinen M, Harma M, Porkka-Heiskanen T, Alenius H (2009). Sleep restriction increases the risk of developing cardiovascular diseases by augmenting proinflammatory responses through IL-17 and CRP. PLoS ONE.

[b34] Maret S, Dorsaz S, Gurcel L, Pradervand S, Petit B, Pfister C, Hagenbuchle O, O’Hara BF, Franken P, Tafti M (2007). Homer1a is a core brain molecular correlate of sleep loss. Proc. Natl Acad. Sci. USA.

[b35] Meisinger C, Heier M, Loewel H (2005). Sleep disturbance as a predictor of type 2 diabetes mellitus in men and women from the general population. Diabetologia.

[b36] Mongrain V, Hernandez SA, Pradervand S, Dorsaz S, Curie T, Hagiwara G, Gip P, Heller HC, Franken P (2010). Separating the contribution of glucocorticoids and wakefulness to the molecular and electrophysiological correlates of sleep homeostasis. Sleep.

[b37] Naidoo N, Giang W, Galante RJ, Pack AI (2005). Sleep deprivation induces the unfolded protein response in mouse cerebral cortex. J. Neurochem.

[b38] Naidoo N, Ferber M, Master M, Zhu Y, Pack AI (2008). Aging impairs the unfolded protein response to sleep deprivation and leads to proapoptotic signaling. J. Neurosci.

[b39] Naidoo N, Zhu J, Zhu Y, Fenik P, Lian J, Galante R, Veasey S (2011). Endoplasmic reticulum stress in wake-active neurons progresses with aging. Aging Cell.

[b40] Ota A, Wang Y (2012). Cdc37/Hsp90 protein-mediated regulation of IRE1alpha protein activity in endoplasmic reticulum stress response and insulin synthesis in INS-1 cells. J. Biol. Chem.

[b41] Oyadomari S, Araki E, Mori M (2002). Endoplasmic reticulum stress-mediated apoptosis in pancreatic beta-cells. Apoptosis.

[b42] Ozcan U, Yilmaz E, Ozcan L, Furuhashi M, Vaillancourt E, Smith RO, Gorgun CZ, Hotamisligil GS (2006). Chemical chaperones reduce ER stress and restore glucose homeostasis in a mouse model of type 2 diabetes. Science.

[b43] Pirot P, Naamane N, Libert F, Magnusson NE, Orntoft TF, Cardozo AK, Eizirik DL (2007). Global profiling of genes modified by endoplasmic reticulum stress in pancreatic beta cells reveals the early degradation of insulin mRNAs. Diabetologia.

[b44] Prinz PN (1995). Sleep and sleep disorders in older adults. J. Clin. Neurophysiol.

[b45] Scheuner D, Vander Mierde D, Song B, Flamez D, Creemers JW, Tsukamoto K, Ribick M, Schuit FC, Kaufman RJ (2005). Control of mRNA translation preserves endoplasmic reticulum function in beta cells and maintains glucose homeostasis. Nat. Med.

[b46] Song B, Scheuner D, Ron D, Pennathur S, Kaufman RJ (2008). Chop deletion reduces oxidative stress, improves beta cell function, and promotes cell survival in multiple mouse models of diabetes. J. Clin. Invest.

[b47] Spiegel K, Knutson K, Leproult R, Tasali E, Van Cauter E (2005). Sleep loss: a novel risk factor for insulin resistance and Type 2 diabetes. J. Appl. Physiol.

[b48] Szegezdi E, Fitzgerald U, Samali A (2003). Caspase-12 and ER-stress-mediated apoptosis: the story so far. Ann. N. Y. Acad. Sci.

[b49] Szegezdi E, Logue SE, Gorman AM, Samali A (2006). Mediators of endoplasmic reticulum stress-induced apoptosis. EMBO Rep.

[b50] Van Cauter E, Holmback U, Knutson K, Leproult R, Miller A, Nedeltcheva A, Pannain S, Penev P, Tasali E, Spiegel K (2007). Impact of sleep and sleep loss on neuroendocrine and metabolic function. Horm. Res.

